# Developmental associations between cognition and adaptive behavior in intellectual and developmental disability

**DOI:** 10.1186/s11689-024-09542-z

**Published:** 2024-06-13

**Authors:** Andrew Dakopolos, Emma Condy, Elizabeth Smith, Danielle Harvey, Aaron J. Kaat, Jeanine Coleman, Karen Riley, Elizabeth Berry-Kravis, David Hessl

**Affiliations:** 1 UC Davis MIND Institute and Department of Psychiatry and Behavioral Sciences, 2825 50th Street, Sacramento, CA 95817 USA; 2 Hofstra University, 1000 Hempstead Tpke, Hempstead, NY 11549 USA; 3 Cincinnati Children’s Hospital Medical Center, 3333 Burnet Ave., Cincinnati, OH 45229 USA; 4 Division of Biostatistics, Department of Public Health Sciences, UC Davis, Medical Sciences 1C, One Shields Avenue, Davis, CA 95616 USA; 5 Feinberg School of Medicine, Northwestern University, 625 N. Michigan Ave Suite 2700, Chicago, IL 60611 USA; 6 Regis University, 3333 Regis Boulevard, Denver, CO 80221 USA; 7 Slippery Rock University of Pennsylvania, 104 Maltby Ave, Slippery Rock, PA 16057 USA; 8 Rush University Medical Center, 600 Paulina St., Chicago, IL 60012 USA

**Keywords:** Cognition, Intellectual and developmental disability, NIH Toolbox, Fragile X syndrome, Down syndrome, Adaptive behavior, Latent change, Structural equation modeling, Longitudinal studies

## Abstract

**Background:**

Intellectual and developmental disabilities (IDDs) are associated with both cognitive challenges and difficulties in conceptual, social, and practical areas of living, commonly referred to as adaptive behavior (DSM–5). Although cross-sectional associations between intelligence or cognition and adaptive behavior have been reported in IDD populations, no study to date has examined whether developmental changes in cognition contribute to or track with changes in adaptive behavior. The present study sought to examine associations of longitudinal developmental change in domains of cognition (NIH Toolbox Cognition Battery, NIHTB-CB) and adaptive behavior domains (Vineland Adaptive Behavior Scales-3; VABS-3) including Socialization, Communication, and Daily Living Skills (DLS) over a two year period in a large sample of children, adolescents and young adults with IDD.

**Methods:**

Three groups were recruited, including those with fragile X syndrome, Down syndrome, and other/idiopathic intellectual disability. Eligible participants (*n* = 263) included those who were between 6 and 26 years (m_age_ = 15.52, sd = 5.17) at Visit 1, and who had a diagnosis of, or suspected intellectual disability (ID), including borderline ID, with a mental age of at least 3.0 years. Participants were given cognitive and adaptive behavior assessments at two time points over a two year period (m = 2.45 years, range = 1.27 to 5.56 years). In order to examine the association of developmental change between cognitive and adaptive behavior domains, bivariate latent change score (BLCS) models were fit to compare change in the three cognitive domains measured by the NIHTB-CB (Fluid Cognition, Crystallized Cognition, Total Cognition) and the three adaptive behavior domains measured by the VABS-3 (Communication, DLS, and Socialization).

**Results:**

Over a two year period, change in cognition (both Crystallized and Total Composites) was significantly and positively associated with change in daily living skills. Also, baseline cognition level predicted growth in adaptive behavior, however baseline adaptive behavior did not predict growth in cognition in any model.

**Conclusions:**

The present study demonstrated that developmental changes in cognition and adaptive behavior are associated in children and young adults with IDD, indicating the potential for cross-domain effects of intervention. Notably, improvements in DLS emerged as a primary area of adaptive behavior that positively related to improvements in cognition. This work provides evidence for the clinical, “real life” meaningfulness of changes in cognition detected by the NIHTB-CB in IDD, and provides empirical support for the NIHTB-CB as a fit-for-purpose performance-based outcome measure for this population.

**Supplementary Information:**

The online version contains supplementary material available at 10.1186/s11689-024-09542-z.

## Background

Intellectual and developmental disabilities (IDDs) are associated with both cognitive challenges and difficulties in conceptual, social, and practical areas of living (DSM–5) [1]. Those with IDD typically present with deficits in intellectual functioning including reasoning, problem solving, planning, abstract thinking, judgment, academic learning, and learning from experience, as well as deficits in adaptive behavior that interfere with independence and living skills within the context of social and cultural developmental norms and expectations [1]. Adaptive behavior refers to one’s ability to function independently across home and community contexts throughout the lifespan [2] and in many individuals with IDD, is an important measure of well-being [3–6]. There is evidence that adaptive behaviors may also serve as a better marker of overall functioning within one’s environment than intellectual ability alone for this population [3, 4].

There is abundant and well characterized cross-sectional [7, 8] and longitudinal [9–12] evidence of deficits in cognition [13–17] and adaptive behavior [9, 18–20] among specific etiologies within IDD (i.e., fragile X syndrome [FXS], Down syndrome [DS], and Williams syndrome [WS]) in development. This strong foundation in the literature has identified phenotypic patterns of relative strengths and weaknesses in cognitive and adaptive domains across people with IDD over time. In particular, Hahn et al. (2015) assessed the effect of non-verbal cognition (measured by the Mullen Scales of Early Learning taken at baseline) on the rate of growth (slopes) and starting level (intercepts) of adaptive behavior subscales in children with FXS between 3 and 6 times from ages 2 to 10 years. They found that non-verbal cognition significantly predicted the rate of growth in daily living skills and motor domains, and significantly predicted the starting level in socialization, communication, and motor domains [11].

In another study that also utilized cross-domain coupling (i.e., baseline intercept of one domain predicting the slope/trajectory of another domain), intelligence (using standard scores from the Kaufman Brief Intelligence Test, 2nd Edition) and adaptive behavior (using the VABS, Second Edition) were examined longitudinally in a sample of children with WS between the ages of 14 and 49 years. In general, intelligence remained stable while adaptive behavior standard scores decreased over time [10]. Baseline intelligence was correlated with a higher intercept (i.e., starting level) of adaptive behavior across all domains (i.e., communication, daily living, socialization), however higher baseline intelligence also predicted greater decreases in adaptive behavior composite and communication scores over time [10].

Despite a robust body of research that has examined adaptive behavior and cognition in IDDs – including those that have investigated both within the same study [21–24], the extent to which change in cognition is related to change in adaptive behavior in individuals with IDDs has not been investigated in any study to our knowledge. It is critically important to characterize how these two broad domains track *within* people with IDD over time as they are core symptom domains in this population across the lifespan. Moreover, understanding both the direction and patterns of association between cognition and adaptive behavior is especially relevant to educational, behavioral, and pharmacological interventions. Specifically, there is great utility in investigating whether improvements in particular cognitive skills – measured by performance-based tests – that may be targeted in treatment, track with clinically meaningful improvements in adaptive behavior. As cognitive tests are increasingly utilized as key performance-based clinical outcome assessments (what the Food and Drug Administration [FDA] refers to as a “PerfO” [25]) in clinical trials and other treatments for people with IDD, it is necessary to understand how changes in cognition as measured by such instruments may associate with and perhaps impact adaptive or functional changes in the individual’s daily life.

The NIH Toolbox Cognition Battery (NIHTB-CB) offers an efficient and standardized approach to capture specific cognitive skills of individuals with IDD as a performance-based measure. Our psychometric studies of the NIHTB-CB in children, adolescents, and young adults with IDD have demonstrated its feasibility and validity [7, 26], as well as its sensitivity to developmental change [12] among this population. In addition, the NIHTB-CB includes tests that target specific cognitive skills including 1) inhibitory control (Flanker Inhibitory Control and Attention [FICA]); 2) working memory (List Sort Working Memory [LSWM]); 3) cognitive flexibility (Dimensional Change Card Sorting [DCCS]); 4) processing speed and attention (Pattern Comparison Processing Speed [PCPS]); 5) memory (Picture Sequence Memory [PSM]); 6) vocabulary (Picture Vocabulary Test [PVT]), and 7) reading/decoding (Oral Reading Recognition Test [ORRT]); all of which are then combined into a Fluid Cognition Composite (1–5), a Crystalized Cognition Composite (6–7), and a Total Cognition Composite (1–7) [7]. In addition to our work with the NIHTB-CB in IDD [7, 12, 26], components of the NIHTB-CB [27] detected treatment effects in a 24-week phase 2 randomized, placebo-controlled, crossover trial of a phosphodiesterase-4D allosteric inhibitor (BPN14770) in 30 adult males with FXS [28]. In this study, compared to placebo, cognitive improvement in BPN14470-treated patients was detected by the language-based NIHTB-CB Crystallized Cognition Composite score.

Multiple active and prospective clinical trials continue to build upon this important work. However, a critical question remains unanswered: Do improvements in cognitive skills, as measured by such tests, *extend to adaptive improvements* in the everyday lives of people with ID? Whether or not improvements in outcomes (i.e., cognitive skills from the NIHTB-CB) translate into identifiable and clinically significant improvements in downstream areas of functioning (such as academic skills, activities of daily living, communication, or social skills) are critically important to our understanding of developmental courses in IDD and will likely be a key determinant for clinical trials moving forward including ultimate FDA acceptance of key outcome measures and eventual targeted treatment approval decisions.

## Methods

### Participants

Eligible participants for this multisite longitudinal study included those who were between 6 and 26 years at Visit 1, and who had a diagnosis of, or suspected IDD. During Visit 1, ID or borderline ID criteria were based on the DSM-5 [1], with adaptive behavior deficits measured by the VABS-3 [2] and IQ < 80 on the Stanford-Binet Intelligence Scales, 5th Edition (SB5). Three groups were recruited: FXS (full mutation, with genetic confirmation), DS (with genetic confirmation if possible), and those with other or idiopathic intellectual disability (OID; with genetic confirmation of negative fragile X mutation). The DS and FXS groups were chosen specifically for this project because of the active translational research programs for these conditions and the urgent need for performance-based outcome measures. The OID group was recruited as an ID comparison group for previously published work [7, 12]. Though we have analyzed NIHTB-CB performance at the group level in these previous investigations, we chose to combine analyses across all groups in the present study given general developmental improvements observed in the NIHTB-CB across all groups previously [12], and to limit the number of analytic models (with multiple reference groups).

A mental age equivalence of at least 3.0 years as measured by the SB5 was required for inclusion, in concordance with NIHTB-CB age limits. Participants were required to be stable with usual treatment for at least 4 weeks before each visit. Exclusion criteria consisted of uncorrectable or uncorrected vision impairment*,* significant motor impairment preventing touch screen or keypad responses*,* or history of head injury, brain infection, stroke, or other neurological problems such as uncontrolled daily seizures or excessive sedation from medication. Recruitment sources consisted of research registries, flyers at local clinics, announcements through parent support foundation websites, and mailings to families registered with state departments that provide services to individuals with IDD. A total of 318 participants with IDD were recruited for Visit 1, and of those recruited, 55 individuals were ineligible: 21 with IQ > 79 and 34 with mental age below 3.0 years, leaving a final sample of 263. Participant retention at visit 2 was 81.36% (*n* = 214, mean_age_ = 17.90 years). Full protocol, details of the NIHTB-CB, and its performance at baseline in the present IDD samples has been reported previously [7, 12, 26].

### Protocol

The NIHTB-CB, VABS-3 interview and SB5 were completed at Visit 1. For some participants, assessments were conducted over two days. After completion of the SB5, participants completed the NIHTB-CB while their parent/caregiver completed the VABS-3 with a psychologist or trained personnel. The same procedure was conducted again approximately 2 years later at Visit 2.

### Measures

The NIHTB-CB [29] (Version 2.0) is an iPad-based battery that assesses Fluid Cognition, Crystalized Cognition, and a Total Cognition Composite through 7 tests as described above in the Introduction. A published manual of standardized NIHTB-CB administration procedures for IDD is available [30]. Unadjusted standard scores (USSs; non age-adjusted) were used for all NIHTB-CB tests. USSs have a mean of 100 and SD of 15. The USSs are recommended for longitudinal measurement because, like change sensitive or growth scale scores, they scale performance across all individuals on the measure based on the difficulty of items they received and their performance relative to everyone else in the original norming sample, allowing scores to be compared across time [31].

The VABS-3 [2] interview form was used to measure adaptive behavior (AB) domains including Communication (consisting of Expressive Language, Receptive Language, and Written Language), Daily Living Skills (DLS; consisting of Personal, and Community skills), and Socialization (consisting of Interpersonal Relationships, Play and Leisure, and Coping Skills). VABS-3 growth scale values (GSVs) were used for all analyses as they have been shown to be sensitive in individuals with IDD, particularly given their robust performance longitudinally and being less susceptible to floor effects [32, 33].

The SB5, which is standardized for individuals between 2–85 years, provides an overall index of intellectual ability reported as the Full-Scale IQ (FSIQ). In part, due to its broad developmental range, the SB5 has performed well in our prior studies of IDD [7, 26, 34]. Our protocol utilizes mental (rather than chronologic) age to select some NIHTB-CB test versions (e.g., PSM) and VABS-3 start points, which were derived from the SB5 FSIQ [30] for each participant. Deviation IQ scores were used to avoid inaccurate assessments of intelligence that can occur with standard score flooring in persons with IDD [34].

### Statistical analyses

Our previous work has identified some limitations using the NIHTB-CB in individuals with IDDs, particularly for those with lower mental ages (i.e., < 5 years) [7]. A specific limitation relates to composite scores in the NIHTB-CB. For instance, the Fluid Cognition Composite is comprised of five subtests, all of which are required to generate a composite score. Many individuals in our sample are unable to pass practice and thus complete all five subtests. Therefore, they do not have composite scores available, thus limiting our power to test hypotheses at the construct level. Structural equation modeling (SEM) provides an analytic framework to help combat this issue, given that this modeling approach is robust to missing data, aiding our ability to retain the full sample in our models. In the present study, bivariate latent change score (BLCS) models provided two-year estimates of both cognitive and adaptive behavior change in individuals with FXS, DS, and OID. This modeling framework allowed us to examine the association between latent change for cognition and adaptive behavior across construct levels of each assessment.

In order to examine the association of developmental change between cognitive and adaptive behavior domains, permutations of BLCS models were used to compare change in the three cognitive domains measured by the NIHTB-CB (Fluid, Crystallized, Total Cognition Composites) and the three AB domains measured by the VABS-3 (Communication, DLS, and Socialization), resulting in the evaluation of nine models plus one full model (including all cognitive domains and all AB domains). Latent change score models are a type of structural equation modeling that provide estimates of change as latent variables based on two or more time points. In the BLCS framework, each model can assess the association between the latent change estimated for two constructs of interest [35]. We have utilized latent change scores previously to characterize developmental change in this sample across individual NIHTB-CB subtests [12]. Missing data were handled with full information maximum likelihood estimation, which is a standard recommendation to provide accurate parameter estimates in the presence of missing data [36].

Generally, each model contained latent scores for cognition and AB at Visit 1 and Visit 2 that were derived from the observed scores from each domain’s respective subtests [35, 37–40]. Latent change scores for cognition (ΔCOG) and adaptive behavior (ΔAB) were included to model change from Visit 1 to Visit 2. Furthermore, we included an estimate of the correlated change between ΔAB and ΔCOG in each model to assess cross-domain coupling of cognition and adaptive behavior. Time between visits and participant age were each used as covariates at the latent level in all models to control for any differences in cognitive and AB change due to variations in timing between Visit 1 and Visit 2, as well as age-related changes – modeled as those between 6 and 16 years at Visit 1, and those 16 years or older at Visit 1, which we have previously demonstrated in this population [12]. Analyses included all participants with a valid NIHTB-CB score, even without completion of visit 2 as BLCS models are robust to missingness [35]. Supplementary Figure 1 graphically presents a generic representation of these models, and Table 3 provides details of each model’s specification. For model fit we first specified base models in which nothing was correlated, and each variable received an equated intercept and variance across time [41]. We then assessed each model’s fit by comparing to its base model (utilizing robust fit parameters including CFI, TLI, and RMSEA) using methods from Savalei et al. [42], and indices of fit based on Little [43] [i.e., CFI > 0.85; TLI > 0.85; RMSEA < 0.08]. In order to control for false discovery rates, Benjamini–Hochberg procedures [44] were conducted for every class of analysis for the models with adequate fit.

## Results

### Descriptive statistics

A sample of 263 individuals were included in the present analyses. Descriptive statistics for sex assigned at birth, race, ethnicity, and diagnostic group are provided in Table 1 and for cognitive and adaptive behavior scores at Visit 1 in Table 2.

**Table 1:** Descriptive statistics for participants at each visit

	**Visit 1**	**Visit 2**
**Sex**	**Percentage (n)**	**Percentage (n)**
Female	40.7 (107)	40.2 (86)
Male	59.3 (156)	59.8 (128)
**Race**		
American Indian/Alaskan Native	1.1 (3)	0.9 (2)
Asian	2.3 (6)	2.8 (6)
Native Hawaiian or Other Pacific Islander	1.1 (3)	0.9 (2)
Black or African American	10.3 (27)	8.9 (19)
White	69.6 (183)	71.5 (153)
More than one race	12.2 (32)	11.7 (25)
Unknown/not reported	3.4 (9)	3.3 (7)
**Ethnicity**		
Hispanic/Latinx	18.3 (48)	17.3 (37)
Not Hispanic/Latinx	77.6 (204)	79.0 (169)
Unknown/not reported	4.2 (11)	3.7 (8)
**Diagnosis**		
Idiopathic/other intellectual disability	33.1 (87)	29.9 (64)
Fragile X syndrome	31.2 (82)	33.6 (72)
Down syndrome	35.7 (94)	36.4 (78)

**Table 2:** Descriptive statistics for study variables (Visit 1)

	**Mean**	**SD**	**Missing (n)**
Chronological age (years)	15.52	5.17	0
SB5 Full Scale mental age* (years)	4.83*	2.12*	0
SB5 Full Scale deviation IQ	53.52	16.31	0
SB5 Nonverbal deviation IQ	55.59	15.19	0
SB5 Verbal deviation IQ	51.45	19.30	0
Vineland-3 ABC	52.60	17.01	16
			**Percent Valid**
NIHTB-CB DCCS^1^	65.60	22.19	60.4 (n = 160)
NIHTB-CB FICA^1^	66.29	24.02	77.7 (n = 206)
NIHTB-CB PVT^2^	67.82	13.40	97.0 (n = 257)
NIHTB-CB PSM^1^	84.50	16.09	89.8 (n = 238)
NIHTB-CB PCPS^1^	70.63	20.62	78.1 (n = 207)
NIHTB-CB ORRT^2^	74.51	14.65	94.0 (n = 249)
NIHTB-CB LSWM^1^	63.99	15.08	54.7 (n = 145)

*DCCS* Dimensional Change Card Sorting, *FICA* Flanker Inhibitory Control and Attention, *PVT* Picture Vocabulary Test, *PSM* Picture Sequence memory, *PCPS* Pattern Comparison Processing Speed, *ORRT* Oral Reading Recognition Test, *LSWM* List Sorting Working Memory, *ABC* Adaptive Behavior Composite, *SB5* Stanford Binet Intelligence Scale, Fifth Edition

^*^Non-normal distribution, median and IQR are reported

^1^indicates NIHTB-CB Fluid Composite subtest

^2^indicates NIHTB-CB Crystallized Composite subtest

### Bivariate latent change score model fit evaluation

Twelve bivariate latent change score models were conducted assessing the relationship between change in the adaptive behavior (ΔAB) domains and change in the cognition (ΔCOG) domains from Visit 1 to Visit 2. The interval between Visit 1 and Visit 2 in years (*M* = 2.45, *SD* = 0.81) was included as a covariate at the latent level, and age, split into those between 6 and 16 years, and 16 years or older at Visit 1 was included as a covariate at the Visit 1 level, as well as the latent level. A model fit statistics summary is presented in Table 3.

**Table 3:**
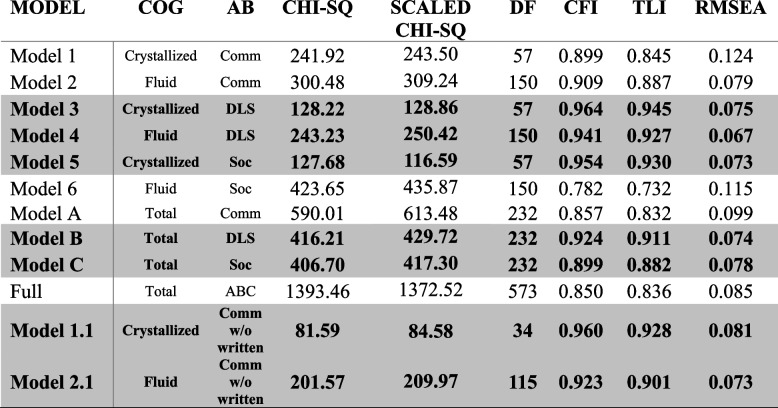
Model fit statistics summary

Shaded models determined to have adequate fit. Goodness of fit according to Little (2013) [43]:

RMSEA: < 0.01 = great, 0.05–0.01 = good, 0.08–0.05 = acceptable, 0.10–0.08 = mediocre, > 0.10 = poor

CFI: > 0.99 = great, 0.95–0.99 = good, 0.90–0.95 = acceptable, 0.85–0.90 = mediocre, < 0.85 = poor

TLI: > 0.99 = great, 0.95–0.99 = good, 0.90–0.95 = acceptable, 0.85–0.90 = mediocre, < 0.85 = poor

*AB* Adaptive Behavior, *Comm* Communication, *Soc* Socialization, *DLS* Daily Living Skills, *ABC* Adaptive Behavior Composite

The first six models (Models 1–6) assessed ΔAB, where AB was modeled as one of three VABS-3 subscales: Communication, DLS, and Socialization, and ΔCOG, where COG was modeled as one of two NIHTB-CB composites (Fluid and Crystallized). Of these models, Model 1 (Crystallized COG and AB Communication) and Model 2 (Fluid COG and AB Communication) demonstrated relatively poor model fit. These models were followed up with Models 1.1 and 2.1, which omitted Written Communication from the AB Communication domain, and were subsequently found to have good fit. Model 6 (Fluid COG and AB Socialization) did not demonstrate adequate model fit, and was not further evaluated. Models 3 (Crystallized COG and AB DLS), 4 (Fluid COG and AB DLS), and 5 (Fluid COG and AB Socialization) were deemed to have good fit.

**Fig. 1:**
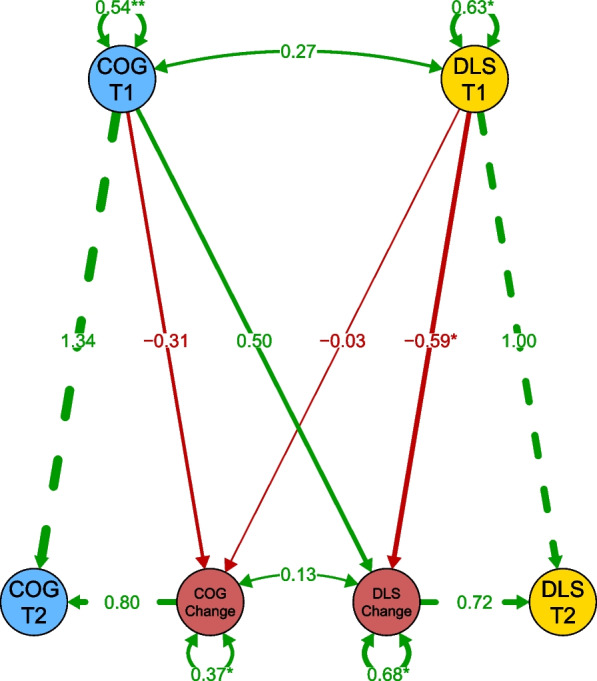
Structural equation model diagram of representative Model B showing association between latent constructs of total cognition composite (COG; NIH Toolbox Cognition Battery) and adaptive behavior (AB; Vineland-3 Daily Living Skills [DLS]) at Visits 1 and 2 and latent change of these constructs across 2 years of development in youth with IDD. Manifest variables omitted for visual clarity

The next set of models (Models A-C) assessed ΔAB across the three subscales of the VABS-3 (Communication, DLS, and Socialization) and ΔCOG across the full NIHTB-CB (comprised of its seven subtests). Of these models, Models B (Total COG and AB DLS) and C (Total COG and AB Socialization) were deemed to have acceptable fit. See Fig. 1 for a graphical representation of the SEM for Model B (Total COG and AB DLS).

A final model (Full Model) assessed ΔAB across the VABS-3 (all domains) and ΔCOG across the full NIHTB-CB. The model fit was poor and not examined further. Notably, none of the models where AB was modeled using the VABS-3 Communication subscales were shown to have good fit until removing the Written Communication subdomain. However, for the other two AB domains (DLS & Socialization), models wherein COG was defined as either Crystallized, Fluid, or the Total NIHTB-CB were shown to have good fit.

### Correlation between cognitive and adaptive behavior change

**Fig. 2:**
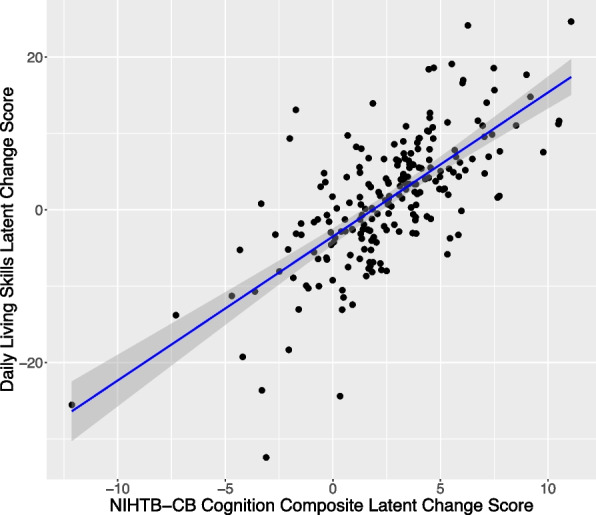
Linear association (with SE shaded) between latent change scores for VABS-3 Daily Living Skills (y-axis) and NIHTB-CB Cognition Composite (x-axis) for Model B

In two of the seven models of good fit, a significant relationship was observed between the change in cognition (ΔCOG) and change in adaptive behavior (ΔAB). A positive relationship between the ΔCOG and ΔAB was observed in Model 3 (Crystallized COG and AB DLS), where COG was a variable comprised of the NIHTB-CB subscales in the Crystallized domain (PVT and ORRT) and AB was comprised of the VABS-3 subscales in the DLS domain (Personal, Domestic, and Community). Similarly, a positive relationship between ΔCOG and ΔAB was observed in Model B (Total COG and AB DLS), where AB was again comprised of the VABS-3 subscales in the DLS domain, but COG was comprised of all of the NIHTB-CB subscales (Fig. 2). These findings indicate that change in cognition, specifically in the Crystallized domain, relates to change in daily living skills over time in our sample. A summary of the parameters of interest from these models is provided in Table 4.

**Table 4:**
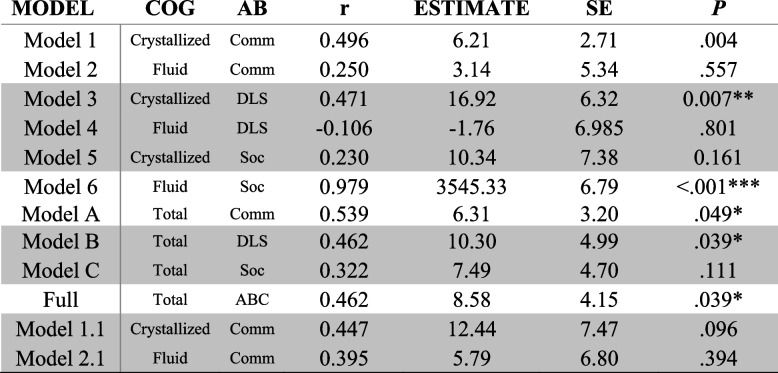
Correlation of latent change (ΔAB and ΔCOG)

Significant paths are denoted by *p < 0.05, **p < 0.01, ***p < 0.001. Shaded rows denote models with good fit

*AB* Adaptive Behavior, *Comm* Communication, *Soc* Socialization, *DLS* Daily Living Skills, *ABC* Adaptive Behavior Composite

### Visit 1 Cross-domain coupling of AB and COG

Tables 5 and 6 present regression parameters for COG at Visit 1, and AB at Visit 1 respectively predicting ΔCOG and ΔAB. Regression components of the models with good fit (i.e., Models 3, 4, 5, B, C, 1.1 and 2.1) were evaluated to examine the influence of COG at Visit 1 on ΔAB and ΔCOG (Table 5), and AB at Visit 1 on ΔAB and ΔCOG (Table 6).

**Table 5:**
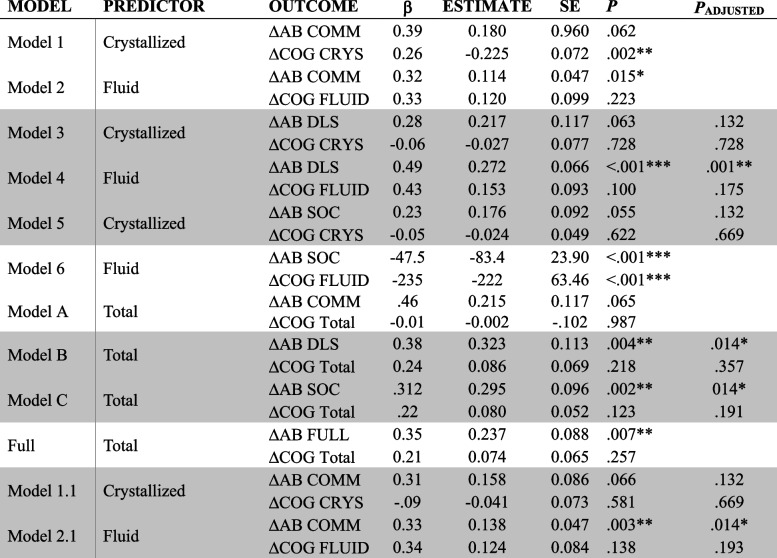
Cross-domain coupling of cognition at Visit 1 and ΔAB and ΔCOG

Significant paths are denoted by *p < 0.05, **p < 0.01, ***p < 0.001. Shaded rows denote models with good fit. *P*_*ADJUSTED*_ utilized Benjamini–Hochberg procedures to control false discovery rate

*AB* Adaptive Behavior, *Comm* Communication, *Soc* Socialization, *DLS* Daily Living Skills, *ABC* Adaptive Behavior Composite

**Table 6:**
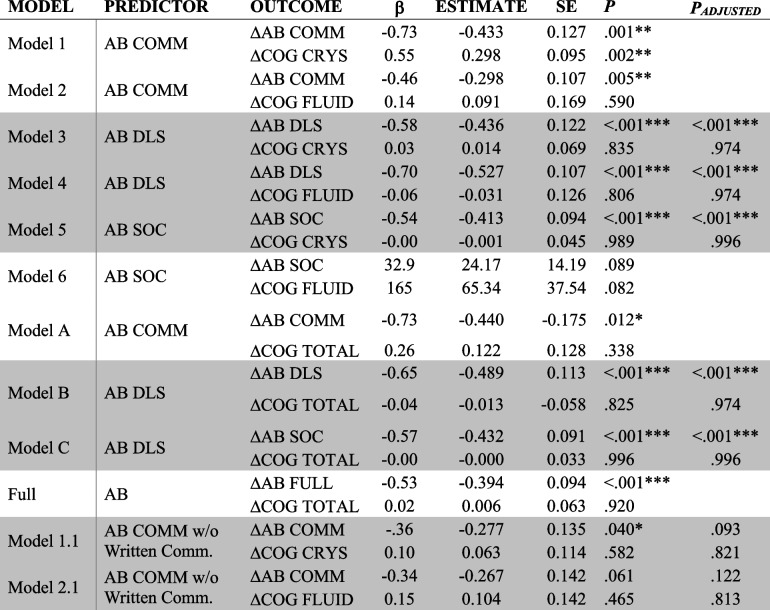
Cross-domain coupling of AB at Visit 1 and ΔAB and ΔCOG

Significant paths are denoted by *p < 0.05, **p < 0.01, ***p < 0.001. Shaded rows denote models with good fit. *P*_*ADJUSTED*_ utilized Benjamini–Hochberg procedures to control false discovery rate

*AB* Adaptive Behavior, *Comm* Communication, *Soc* Socialization, *DLS* Daily Living Skills, *ABC* Adaptive Behavior Composite

COG at Visit 1 significantly predicted increased developmental change in AB for Models 4 (Fluid COG and AB DLS), and 2.1 (Fluid COG and AB Communication without Written Language), as well as Models B (Total COG and AB DLS) and C (Total COG and AB Socialization), however COG at Visit 1 did *not* predict ΔCOG in any model. This pattern of results indicates that Crystallized Cognition, Fluid Cognition and the Total Cognition Composites are good indicators of developmental change in daily living skills, socialization, and expressive/receptive language; however individual starting cognition scores are not predictive of individuals’ subsequent cognitive development.

For the cross-domain coupling of AB at Visit 1 on ΔCOG, AB at Time 1 did *not* predict ΔCOG in any model. AB at Visit 1 predicted less change in ΔAB for all models (i.e., Models 3, 4, 5, 1.1, B, and C) indicating that those with higher DLS, Socialization, and Expressive/Receptive Communication scores at Visit 1 reported *less* improvement in those respective adaptive behavior skills after two years.

## Discussion

The present study examined the association between developmental change in cognition and adaptive behavior in children, adolescents and young adults with IDD. We modeled this association using bivariate latent change score models. We found that developmental improvements in language-based crystallized cognition and overall cognition as measured by the NIHTB-CB were related to improvements in daily living skills. Models that included the VABS-3 Communication domain (i.e., Models 1, 2, A, and the Full Model) did not have adequate model fit to report reliable results. Follow-up analyses indicated that the measurement model for VABS-3 Communication did not fit due to a high degree of written communication covariance with the other VABS-3 Communication areas (i.e., Receptive Communication and Expressive Communication). Models excluding Written Communication were subsequently fit (Model 1.1, 2.1).

The present study is the first, to our knowledge, to assess the relation between developmental change in cognition and adaptive behavior in individuals with IDD, the two central domains of functioning for this population. The results may indicate the potential for cross-domain effects of intervention. Notably, improvements in daily living skills emerged as a primary area of adaptive behavior that positively related to improvements in cognition. In addition, cognitive skills at Visit 1 predicted developmental change in all domains of adaptive behavior after an average of a little over two years; specifically fluid cognition at Visit 1 predicted improvements in daily living skills and communication, and overall cognition at Visit 1 predicted improvement in daily living skills and social skills.

These findings demonstrate some of the “real-life” improvements that are associated with, and perhaps in part driven by cognitive growth in youth with IDD. Previous work has shown that cognitive ability, as measured by IQ testing, is correlated with adaptive functioning [45] at the population level. With the strength of this relationship in mind, it begs the question whether treatment-related improvements in cognition may accelerate the development of adaptive skills. Establishing such reciprocity has implications for how treatment trials in IDD are designed and implemented [10, 21, 46]. However, the present study cannot draw any causal inferences as it was correlational. Future innovative controlled, randomized clinical trials [47, 48], examining the impact of cognition-enhancing medications or behavioral interventions (e.g., to improve flexibility, problem-solving, and executive function), could be fruitful.

The importance of developing endpoint measures in the field of IDD is evident [49], with many existing measures of the concept of interest (i.e., cognition) deemed inadequate or not fully “fit for purpose.” Measures commonly used in clinical trials have been critiqued for their limitations in validation, sensitivity to change, or clinical meaningfulness in individuals with IDD [50]. Historically, when evaluating clinical outcome assessments (COAs) for IDD, PerfO’s (i.e., direct assessments), have excluded intelligence tests as they have been considered inappropriate for shorter-term interventions because they tap “stable” traits that are less likely to show dynamic changes during these time frames; thus, COAs for IDD often consist of observer ratings or clinician reported outcomes [51]. The NIHTB-CB was developed, in part, with the express purpose of filling the performance outcome gap for cognition in intervention studies, and it includes tests of fluid cognitive constructs that may be more sensitive to interventions. However, it was not created, validated, or normed with consideration of IDD, a population currently undergoing clinical trials targeting cognition and in urgent need of suitable primary outcome measures. Nonetheless, validity evidence for the NIHTB-CB has been collected in IDD [7] and it shows sensitivity to change in this population [12]. Evaluating the clinical meaningfulness of the NIHTB-CB was an important next step. The present study established the clinical meaningfulness of the NIHTB-CB through its relation to adaptive behaviors. Note that while the VABS-3 is often used as an outcome measure in clinical trials for ID, it is not a direct assessment (it is a combination of an observer report outcome and clinician-reported outcome), perhaps limiting its sensitivity.

The negative association between baseline adaptive behavior level and subsequent change in adaptive skills was a surprising finding. Specifically, higher adaptive behavior skills at the first visit was associated with less growth in adaptive behavior over the following 2 years in all models. Such a pattern could be indicative of a regression to the mean, or evidence that as an individual approaches a skill ceiling, their growth in that domain will begin to slow. Conversely, this finding also indicates that individuals with lower baseline adaptive levels demonstrated, on average, greater rates of growth over two years. Interestingly, this effect was not apparent within the cognitive domain, as cognitive ability at Visit 1 was not significantly associated with change in cognitive ability, only with change in adaptive behavior. The models revealed a positive relationship such that higher cognitive ability at Visit 1 was related to increased growth in adaptive behavior.

Latent change score models were used to resolve issues with missing data and utilized growth scale values (GSVs) to more accurately model change in the present study. Missing data were particularly problematic for NIHTB-CB tests in the Fluid domain, notably Flanker and DCCS. These tasks are challenging for individuals with IDD [7] as well as individuals of young mental ages, including young typically developing children [52]. Unfortunately, missing scores on any individual NIHTB-CB test prohibits the generation of Fluid, Crystallized, and Composite scores. For this reason, large portions of our sample would have been excluded from the analyses if a latent variable approach had not been used to model the cognitive domains. Additionally, VABS-3 GSVs are only available at the subdomain level. GSVs cannot be averaged across subdomains to create composites for Communication, Socialization, and DLS domains because they are “a unitless measure and therefore cannot be compared or combined across subdomains” [53]. By utilizing latent change score models, we were able to use our data in full to examine the broader constructs of cognition and adaptive functioning. However, this approach presents practical challenges. Namely, the latent variable “scores” in these models do not match the composite or domain scores generated by the NIHTB-CB or the VABS-3. For this reason, the correlations between latent variables cannot be translated into practical terms regarding the standard output that are generated by these tests (e.g., “An *x* point change in the NIHTB-CB Crystallized Composite is associated with a *y* point change in the VABS-3 ABC"). The present study instead provides evidence that change in certain domains of cognitive function and adaptive behavior are associated at the latent level.

Related to the data missingness of the NIHTB-CB tests, future development of the NIHTB-CB could involve individuals with IDD in test development and norming to improve feasibility. The development of the NIH Infant and Toddler (Baby) Toolbox (NBT), including domains of cognition and executive function, language, numeracy/early mathematics, motor, and social functioning, is currently underway. The NBT aims to capture neurodevelopment at younger ages (1–42 months old) for both research and clinical use. Individuals with IDD represent a clear clinical population of interest for this measure, particularly due to the much lower mental ages often seen in this population, and the limited feasibility we have observed for some fluid reasoning tests in individuals with these lower mental ages.

We have previously demonstrated that rates of change vary depending on the chronological age of the participants [12]. Even among those with IDD, more change is anticipated at younger ages than at older ages, when growth plateaus (and eventually decreases). As such, we dichotomized age to represent these two groups—those where more change is anticipated versus those where less may be expected. Simply mean-centering age and using it as a continuous variable would fail to account for these different developmental expectations. We chose to dichotomize age into these developmental epochs as age at baseline was not the primary concern for our hypotheses and we wanted to reduce model complexity where possible. Another study caveat pertains to the time span between assessments (slightly over two years on average), and the number of observations across development (maximum of two), factors that likely reduced power to detect associations of cognition and adaptive behavior growth. More observations over a longer period of development would likely produce better and stronger estimates of these associations.

## Conclusions

In summary, the present study demonstrated that cognitive level, as well as change in cognition over a two-year period of development, as measured by the NIHTB-CB, are associated with growth in adaptive behavior, especially daily living skills, among youth with IDD. This work provides evidence for the clinical, “real life” meaningfulness of the NIHTB-CB in IDD, and important empirical support for the NIHTB-CB as a fit-for-purpose performance-based outcome measure for this population.

### Supplementary Information

Supplementary Material 1: Supplementary Figure 1. Structural equation model diagram of the full bivariate latent change score model.

## Data Availability

Data are available from the NIMH Data Archive (nda.nih. gov/)—ID C3738.

## Abbreviations

IDD: Intellectual and developmental disability
DSM: Diagnostic and Statistical Manual of Mental Disorders
NIHTB-CB: National Institutes of Health Toolbox Cognition Battery
VABS-3: Vineland Adaptive Behavior Scales, Third Edition
BLCS: Bivariate latent change score
DLS: Daily Living Skills
FXS: Fragile X syndrome
DS: Down syndrome
WS: Williams syndrome
FDA: Federal Drug Administration
SB5: Stanford Binet Intelligence Scales, Fifth Edition
FICA: Flanker Inhibitory Control and Attention
DCCS: Dimensional Change Card Sort
PCPS: Picture Comparison Processing Speed
LSWM: List Sorting Working Memory
PSM: Picture Sequence Memory
PVT: Picture Vocabulary Test
ORRT: Oral Reading Recognition Test
USS: Unadjusted standard score
AB: Adaptive behavior
CFI: Comparative fit index
RMSEA: Root mean square error of approximation
TLI: Tucker-Lewis index
IQR: Inter-quartile range
COG: Cognition
COA: Clinical outcome assessment
GSV: Growth scale value
ABC: Adaptive Behavior Composite
NBT: National Institutes of Health Baby Toolbox

## References

[1] American Psychiatric Association. Diagnostic and Statistical Manual of Mental Disorders. (American Psychiatric Association, Washington DC. 2013. 10.1176/appi.books.9780890425596.

[2] Sparrow SS, Cicchetti DV, Saulnier CA. Vineland Adaptive Behavior Scales, Third Edition (Vineland^TM^-3) Comprehensive Interview Form Report. 2016.

[3] Bertollo JR, Yerys BE (2019). More than IQ: Executive function explains adaptive behavior above and beyond nonverbal IQ in youth with autism and lower IQ. Am J Intellect Dev Disabil.

[4] Kanne SM (2011). The role of adaptive behavior in autism spectrum disorders: Implications for functional outcome. J Autism Dev Disord.

[5] Hartley SL et al. Exploring the adult life of men and women with fragile X syndrome: Results from a national survey. American Journal on Intellectual and Developmental Disabilities. 2011. 116 16–35 Preprint at 10.1352/1944-7558-116.1.16. 10.1352/1944-7558-116.1.16 PMC3238098 21291308

[6] Elshani H, Dervishi E, Ibrahimi S, Nika A, Maloku Kuqi M (2020). Adaptive Behavior in Children with Intellectual Disabilities. Mediterr J Soc Sci.

[7] Shields RH (2020). Validation of the NIH Toolbox Cognitive Battery in intellectual disability. Neurology.

[8] Will EA, Caravella KE, Hahn LJ, Fidler DJ, Roberts JE (2018). Adaptive behavior in infants and toddlers with Down syndrome and fragile X syndrome. Am J Med Genet B Neuropsychiatr Genet.

[9] Caravella KE, Roberts JE (2017). Adaptive skill trajectories in infants with fragile X syndrome contrasted to typical controls and infants at high risk for autism. Res Autism Spectr Disord.

[10] Fisher MH, Lense MD, Dykens EM. Longitudinal trajectories of intellectual and adaptive functioning in adolescents and adults with Williams syndrome. in Journal of Intellectual Disability Research vol. 60 920–932 (Blackwell Publishing Ltd, 2016). 10.1111/jir.12303 27273269

[11] Hahn LJ, Brady NC, Warren SF, Fleming KK (2015). Do Children With Fragile X Syndrome Show Declines or Plateaus in Adaptive Behavior?. Am J Intellect Dev Disabil.

[12] Shields RH (2023). Sensitivity of the NIH Toolbox to Detect Cognitive Change in Individuals With Intellectual and Developmental Disability. Neurology.

[13] Lukowski AF, Milojevich HM, Eales L. Cognitive Functioning in Children with Down Syndrome: Current Knowledge and Future Directions. in Advances in Child Development and Behavior vol. 56 257–289 (Academic Press Inc., 2019). 10.1016/bs.acdb.2019.01.002 30846049

[14] Onnivello, S. et al. Cognitive profiles in children and adolescents with Down syndrome. Sci Rep. 2022;12. 10.1038/s41598-022-05825-4 PMC8816899 35121796

[15] Razak, K. A., Dominick, K. C. & Erickson, C. A. Developmental studies in fragile X syndrome. Journal of Neurodevelopmental Disorders. 2020. vol. 12 Preprint at 10.1186/s11689-020-09310-9. 10.1186/s11689-020-09310-9 PMC7196229 32359368

[16] Schmitt, L. M., Shaffer, R. C., Hessl, D. & Erickson, C. Executive function in fragile X syndrome: A systematic review. Brain Sciences. 2019 vol. 9 Preprint at 10.3390/brainsci9010015. 10.3390/brainsci9010015 PMC6356760 30654486

[17] Condy EE (2022). NIH Toolbox Cognition Battery Feasibility in Individuals With Williams Syndrome. Am J Intellect Dev Disabil.

[18] Onnivello S. et al. Executive functions and adaptive behaviour in individuals with Down syndrome. in Journal of Intellectual Disability Research. 2022. vol. 66 32–49 (John Wiley and Sons Inc). 10.1111/jir.12897 PMC9299024 34750907

[19] Hatton DD. et al. Adaptive Behavior in Children With Fragile X Syndrome. American Association on Mental Retardation. 2003;373. 10.1352/0895-8017(2003)108<373:ABICWF>2.0.CO;2 14561110

[20] Tomaszewski B, Hepburn S, Blakeley-Smith A, Rogers SJ (2020). Developmental Trajectories of Adaptive Behavior from Toddlerhood to Middle Childhood in Autism Spectrum Disorder. Am J Intellect Dev Disabil.

[21] Klaiman C (2014). Longitudinal profiles of adaptive behavior in fragile X syndrome. Pediatrics.

[22] Hawkins BA, Eklund SJ, James DR, Foose AK (2003). Adaptive behavior and cognitive function of adults with Down syndrome: Modeling change with age. Ment Retard.

[23] Fisch GS, Simensen RJ, Schroer RJ (2002). Longitudinal changes in cognitive and adaptive behavior scores in children and adolescents with the fragile X mutation or autism. J Autism Dev Disord.

[24] Fisch GS. et al. Developmental trajectories in syndromes with intellectual disability, with a focus on wolf-hirschhorn and its cognitive-behavioral profile. American Journal on Intellectual and Developmental Disabilities. 2012. vol. 117 167–179 Preprint at 10.1352/1944-7558-117.2.167. 10.1352/1944-7558-117.2.167 22515830

[25] Walton MK (2015). Clinical Outcome Assessments: Conceptual Foundation-Report of the ISPOR Clinical Outcomes Assessment-Emerging Good Practices for Outcomes Research Task Force. Value Health.

[26] Hessl D. et al. The NIH Toolbox Cognitive Battery for intellectual disabilities: Three preliminary studies and future directions. J Neurodev Disord. 2016;8. 10.1186/s11689-016-9167-4 PMC5012003 27602170

[27] Gershon RC (2010). Assessment of neurological and behavioural function: the NIH Toolbox. Lancet Neurol.

[28] Berry-Kravis EM (2021). Inhibition of phosphodiesterase-4D in adults with fragile X syndrome: a randomized, placebo-controlled, phase 2 clinical trial. Nat Med.

[29] Gershon RC (2013). NIH toolbox for assessment of neurological and behavioral function. Neurology.

[30] Mckenzie F et al. National Institutes of Health Toolbox Cognitive Battery Supplemental Administrator’s Manual for Intellectual and Developmental Disabilities A Guide on Administration and Scoring Standards.

[31] Casaletto KB (2015). Demographically Corrected Normative Standards for the English Version of the NIH Toolbox Cognition Battery. J Int Neuropsychol Soc.

[32] Farmer C, Adedipe D, Bal V, Chlebowski C, Thurm A. Reliability of the Vineland Adaptive Behavior Scales, Third Edition. 10.1111/jir.12691 PMC6941197 31657503

[33] Farmer CA. et al. Person ability scores as an alternative to norm-referenced scores as outcome measures in studies of neurodevelopmental disorders. American Journal on Intellectual and Developmental Disabilities. 2020. vol. 125 475–480 Preprint at 10.1352/1944-7558-125.6.475. 10.1352/1944-7558-125.6.475 PMC11485197 33211814

[34] Sansone SM. et al. Improving IQ measurement in intellectual disabilities using true deviation from population norms. J Neurodev Disord. 2014;6. 10.1186/1866-1955-6-16 PMC4613563 26491488

[35] Kievit RA. et al. Developmental cognitive neuroscience using latent change score models: A tutorial and applications. Developmental Cognitive Neuroscience. 2018. vol. 33 99–117 Preprint at 10.1016/j.dcn.2017.11.007. 10.1016/j.dcn.2017.11.007 PMC6614039 29325701

[36] Widaman KF. Best practices in quantitative methods for developmentalists: III. Missing data: What to do with or without them. Monogr Soc Res Child Dev. 2006. 10.1111/j.1540-5834.2006.07103001.x 17199773

[37] McArdle JJ (2009). Latent variable modeling of differences and changes with longitudinal data. Annu Rev Psychol.

[38] R Core Team, R (2013). R: A language and environment for statistical computing.

[39] Rosseel Y (2012). lavaan: An R package for structural equation modeling. J Stat Softw.

[40] Ghisletta P, McArdle JJ (2012). Latent curve models and latent change score models estimated in R. Struct Equ Modeling.

[41] Widaman KF, Thompson JS (2003). On Specifying the Null Model for Incremental Fit Indices in Structural Equation Modeling. Psychol Methods.

[42] Savalei V (2018). On the Computation of the RMSEA and CFI from the Mean-And-Variance Corrected Test Statistic with Nonnormal Data in SEM. Multivariate Behav Res.

[43] Little TD. Longitudinal Structural Equation Modeling. (Guilford press, 2013).

[44] Benjamini Y, Hochberg Y. Controlling the False Discovery Rate: A Practical and Powerful Approach to Multiple Testing. Source: Journal of the Royal Statistical Society. Series B (Methodological). 1995;57.

[45] Alexander RM, Reynolds MR (2020). Intelligence and Adaptive Behavior: A Meta-Analysis. School Psych Rev.

[46] Esbensen AJ (2017). Outcome measures for clinical trials in down syndrome. Am J Intellect Dev Disabil.

[47] de la Torre R (2016). Safety and efficacy of cognitive training plus epigallocatechin-3-gallate in young adults with Down’s syndrome (TESDAD): A double-blind, randomised, placebo-controlled, phase 2 trial. Lancet Neurol.

[48] Hessl, D. et al. Cognitive training for children and adolescents with fragile X syndrome: a randomized controlled trial of Cogmed. J Neurodev Disord. 2019;11. 10.1186/s11689-019-9264-2 PMC6463634 30982467

[49] Esbensen A, Schworer E. Contemporary Issues in Evaluating Treatment in Neurodevelopmental Disorders. (Elsevier, 2022).

[50] Budimirovic, D. B. et al*.* Updated report on tools to measure outcomes of clinical trials in fragile X syndrome. Journal of Neurodevelopmental Disorders. 2017. vol. 9 Preprint at 10.1186/s11689-017-9193-x. 10.1186/s11689-017-9193-x PMC5467057 28616097

[51] Farmer C, Adedipe D, Bal V, Chlebowski C, Thurm A. Reliability of the Vineland Adaptive Behavior Scales, Third Edition. 10.1111/jir.12691 PMC6941197 31657503

[52] Becker L, Condy E, Kaat A, Thurm A (2023). How do 3-year-olds do on the NIH Toolbox Cognitive Battery?. Child Neuropsychol.

[53] Farmer C, Thurm A, Troy JD, Kaat AJ. Comparing ability and norm-referenced scores as clinical trial outcomes for neurodevelopmental disabilities: a simulation study. J Neurodev Disord. 2023;15. 10.1186/s11689-022-09474-6 PMC9843928 36650450

