# Supplementary figures and images for: Developmental associations between cognition and adaptive behavior in intellectual and developmental disability
Source: J Neurodev Disord. 2024 Jun 13;16:31. doi: 10.1186/s11689-024-09542-z (PMC11177479; doi:10.1186/s11689-024-09542-z)

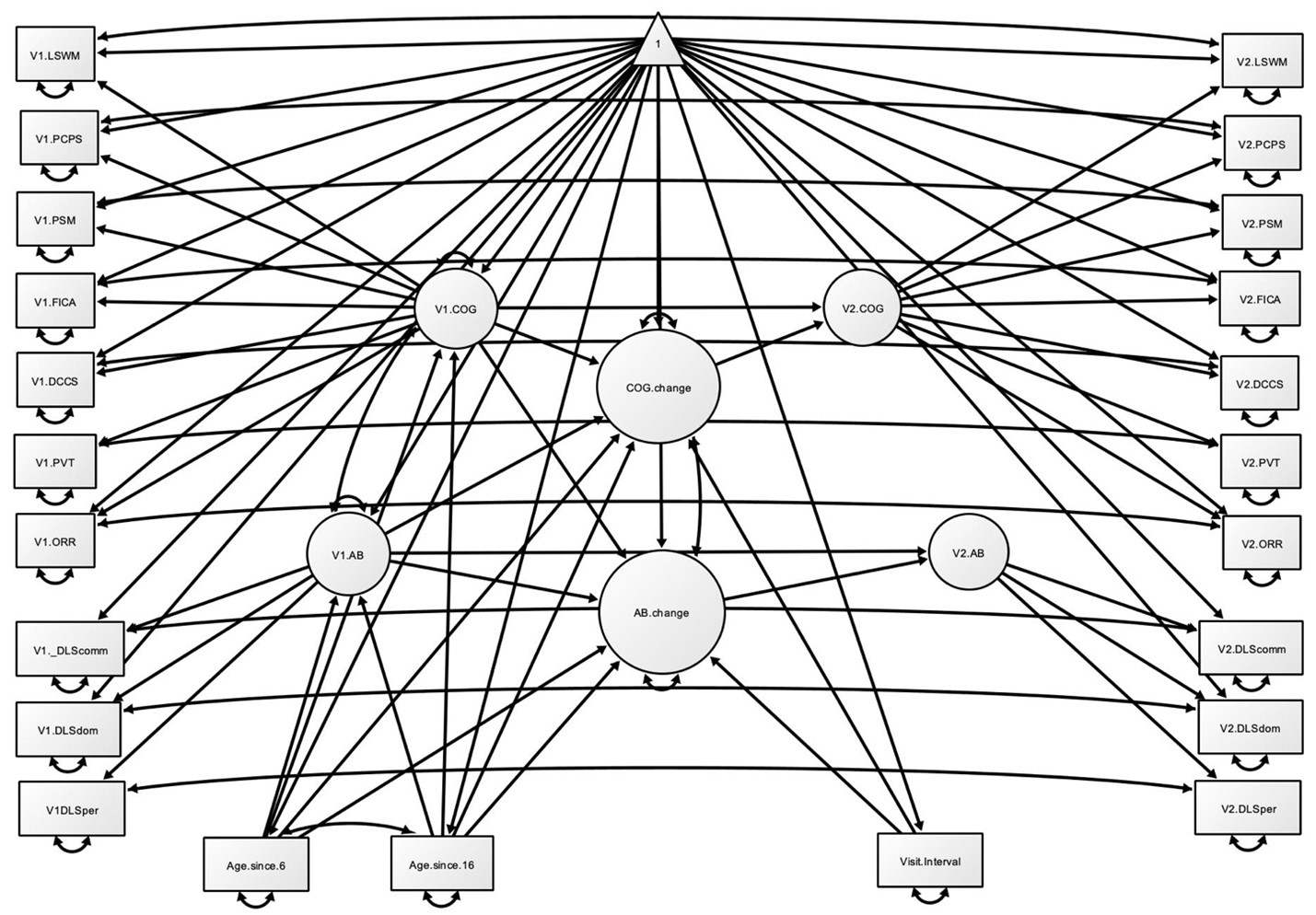

Supplement: Supplementary file 1 — Supplementary Material 1: Supplementary Figure 1. Structural equation model diagram of the full bivariate latent change score model. [file 11689_2024_9542_MOESM1_ESM.jpg]
